# Next-generation visitation models using social media to estimate recreation on public lands

**DOI:** 10.1038/s41598-020-70829-x

**Published:** 2020-09-22

**Authors:** Spencer A. Wood, Samantha G. Winder, Emilia H. Lia, Eric M. White, Christian S. L. Crowley, Adam A. Milnor

**Affiliations:** 1 eScience Institute, University of Washington, Seattle, WA USA; 2 EarthLab, University of Washington, Seattle, WA USA; 3 Pacific Northwest Research Station, US Forest Service, Olympia, WA USA; 4 Office of Policy Analysis, US Department of the Interior, Washington, DC USA; 5 Rivers, Trails, and Conservation Assistance Program, National Park Service, Tucson, AZ USA

**Keywords:** Conservation biology, Ecosystem services

## Abstract

Outdoor and nature-based recreation provides countless social benefits, yet public land managers often lack information on the spatial and temporal extent of recreation activities. Social media is a promising source of data to fill information gaps because the amount of recreational use is positively correlated with social media activity. However, despite the implication that these correlations could be employed to accurately estimate visitation, there are no known transferable models parameterized for use with multiple social media data sources. This study tackles these issues by examining the relative value of multiple sources of social media in models that estimate visitation at unmonitored sites and times across multiple destinations. Using a novel dataset of over 30,000 social media posts and 286,000 observed visits from two regions in the United States, we compare multiple competing statistical models for estimating visitation. We find social media data substantially improve visitor estimates at unmonitored sites, even when a model is parameterized with data from another region. Visitation estimates are further improved when models are parameterized with on-site counts. These findings indicate that while social media do not fully substitute for on-site data, they are a powerful component of recreation research and visitor management.

## Introduction

Public lands provide a broad range of economic, social, and health benefits. Well-managed parks and protected areas are essential places to recreate, exercise, socialize, and experience nature^1^. To maintain the social and conservation benefits of natural areas, public land managers rely on information on the amount and character of visitor use. Additionally, understanding how visitors interact with natural and built environments is critical for managing natural resources and biodiversity, maintaining amenities, and ensuring visitor safety. However, the time and expense required to count and survey visitors results in limited and uneven data availability. Without accurate and current information, decision-makers are challenged to meet the increasing and varying demand for outdoor recreation, evaluate management options, advocate for parks and protected areas, and prioritize spending on public lands^2^.

Many recent studies have proposed that volunteered geographic data from social media can complement existing information about visitor distributions, behaviors, and preferences^3–8^. Studies spanning an impressive diversity of developed and undeveloped settings have concluded that the popularity of parks is generally mirrored in the popularity of the same destinations on individual social media platforms such as Flickr, Instagram, Sina Weibo, and Twitter^9–15^ (but see Ref.^16^). The consensus emerging from these correlational studies is that data from social media have potential to inform estimates of absolute visitation at specific destinations and for multiple time periods^17–19^. To the extent that social media offer additional information across geographic or temporal scales, these data may complement traditional on-site counts in parks and protected areas. Where social media use varies predictably with actual visitation, it may also serve as a substitute for on-site data, particularly in areas considered too costly or difficult for traditional monitoring. Efficiently and accurately estimating absolute numbers of visitors at unmonitored sites and times is, nevertheless, a goal yet to be realized.

Despite its potential, several serious issues may limit the applicability of social media in generalizable and predictive models of park use. Globally, the performance of social media as a visitation proxy varies geographically and by type of attraction^8,12,20^. This may be due to differences in who uses various platforms^21,22^, how users share content^23,24^, and changes in the popularity of platforms over time^15,25,26^. Some studies suggest that these issues may be partly overcome by combining data from multiple social media platforms^4,9,12,17,20,27^. However, data quality and interpretability varies across users and service providers^28^, as do restrictions on which, when, and how data can be accessed^29^. Likewise, the cost and effort required to obtain and prepare data can vary unpredictably from year to year. There are also important ethical, legal, and privacy concerns to consider^30,31^. These issues of representation, data availability, and privacy—which are only beginning to be understood in the context of outdoor recreation—cast some doubt on the practical value of volunteered geographic data for practitioners who seek fine-scale data on visitor use.

This study considers the promise and potential pitfalls of using social media to estimate recreational visitation to sites on public lands. Using a novel dataset of recreation counts derived from on-site surveys and three social media data sources, we develop an empirical approach for modeling visitation in two geographically separate regions, contrasting social media use with observed visitation within and between regions. We evaluate the potential for visitation models created for one region to successfully predict visitation at recreation sites in the other, then discuss the similarities and differences in how social media use is related to visitation at local and large geographic scales. This paper concludes with a discussion of the implications of our results for recreation research and visitor management.

## Results

### Recreation counts

**Figure 1:**
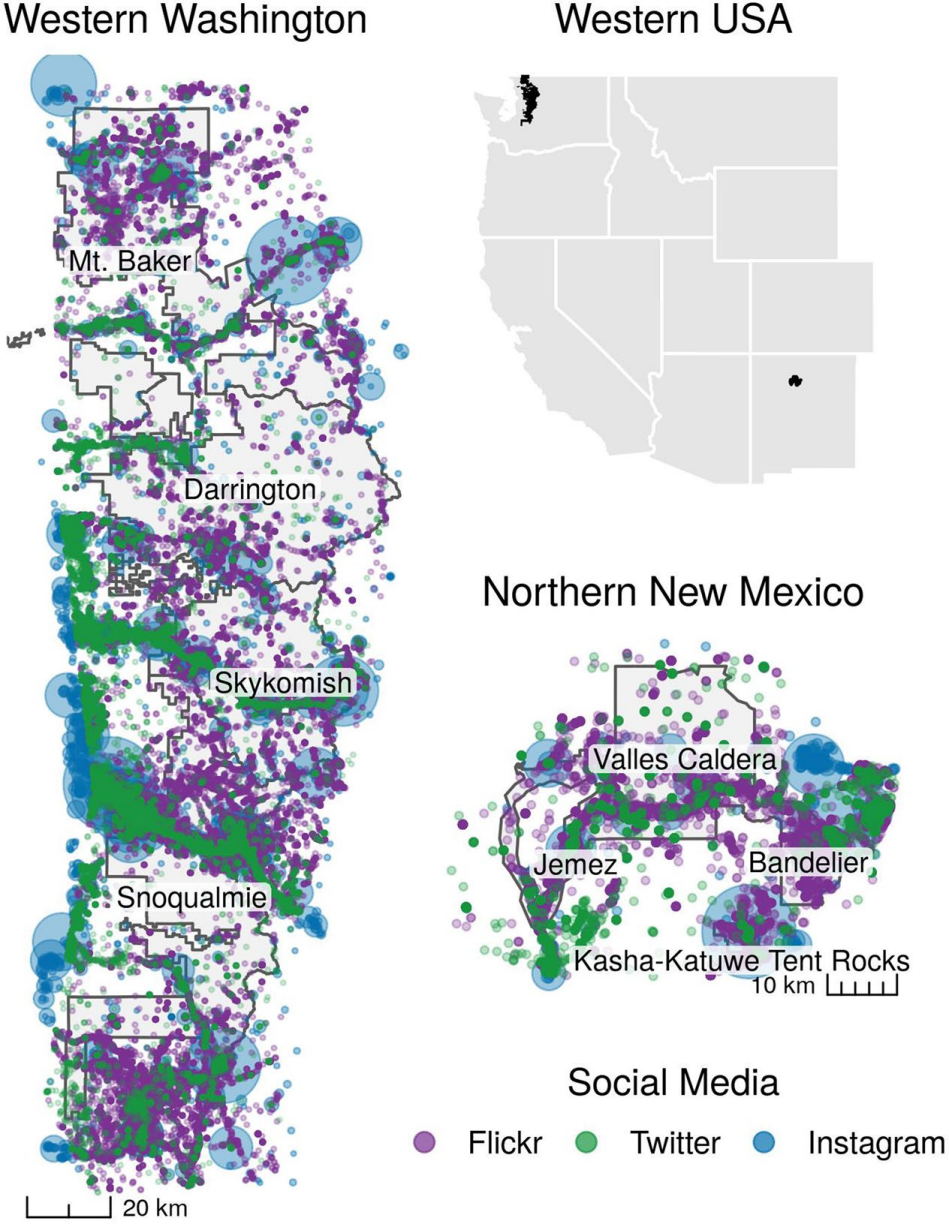
Locations of geotagged social media posts made by visitors to public lands in WWA and NNM. Points represent the latitude and longitude where a Flickr photograph (purple) or tweet (green) was created. For Instagram, points represent places to which images were assigned by users (blue). Larger points represent a greater number of Instagram images from the location. Study sites are contained within the management units (shaded grey). Figure created using R^48^ version 3.5.3.

Our on-site counters logged 286,343 visitor-days of recreational use across 26 sites on public lands in Western Washington (WWA) and 13 sites in Northern New Mexico (NNM) over the same 27-week time period from April 27, 2018 to October 24, 2018 (Fig. 1, Table 1). Each site was defined as a destination where a visitor might recreate for one day or a limited number of days. On-site counts collected in WWA outside the time period of data collection in NNM (Supplementary Fig. S1) were not included in the following comparisons. Observed visitation was highest in WWA between July and September; in NNM it peaked in late May (over Memorial Day weekend) and in early October (coincident with the Albuquerque International Balloon Festival).

**Table 1:** Comparison of visitation and social media use between WWA and NNM (April 27 to October 24, 2018).

Observations		Western Washington		Northern New Mexico	
Sites monitored		26		13	
Site-week observations		390		230	
Total observed visitor-days		196,178		90,165	
Sites with no social media posts		1		6	

Social media		# User-days	% User-days (%)	# User-days	% User-days (%)
Instagram		7,558	3.85	2,723	3.02
Twitter		80	0.04	9	0.01
Flickr		39	0.02	8	0.01

The number of social media user-days was divided by total observed visitor days to calculate percent user-days. Note that several weeks of data were excluded in both regions due to site closures as well as some faulty on-site counting devices.

**Figure 2:**
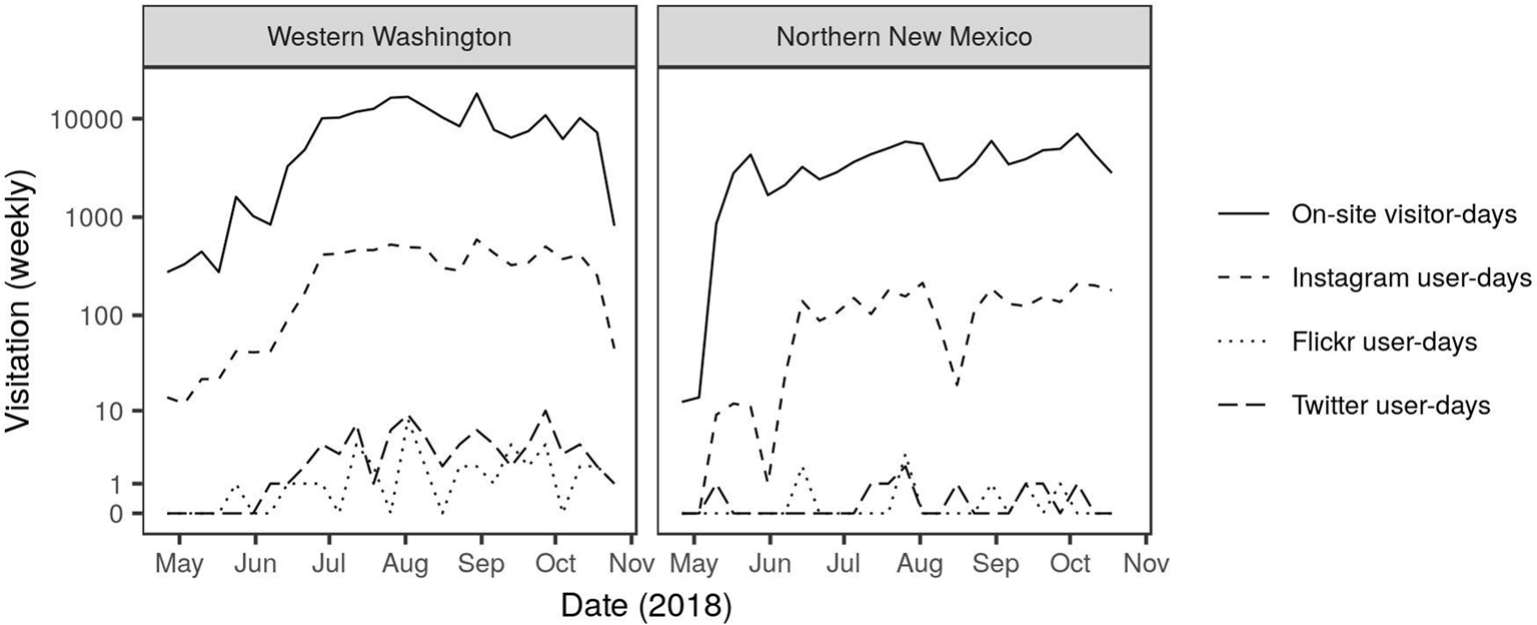
Total weekly visitation (on a log scale) per region for 26 sites in WWA and 13 sites in NNM, between April 27 and October 24, 2018, as measured by on-site counts and social media sources. Note that both Flickr and Twitter consistently measure fewer than ten user-days per week.

The share of visitors posting to social media in 2018 was similar in these two very different regions, as seen by comparing social media user-days against total on-site visitor-days. For both WWA and NNM, the number of posts on Instagram was between 3 and 4% of total observed user-days. Posts on Twitter or Flickr were less than 1% of total observed user-days (Table 1). Over time, Instagram user-days tracked visitation relatively closely in both regions. Flickr and Twitter tracked visitation to some degree, but were limited by the sparseness of posts to these platforms (Fig. 2). Instagram was the most popular platform for sharing social media from the study sites in summer 2018 (10,281 user-days for WWA and NNM combined), while Flickr and Twitter each registered fewer than 100 total user-days of content for these sites (Table 1). Six of the 13 study sites that we observed in NNM registered zero social media user-days according to our methods. In contrast, only one of the 26 study sites in WWA registered zero social media user-days.

**Figure 3:**
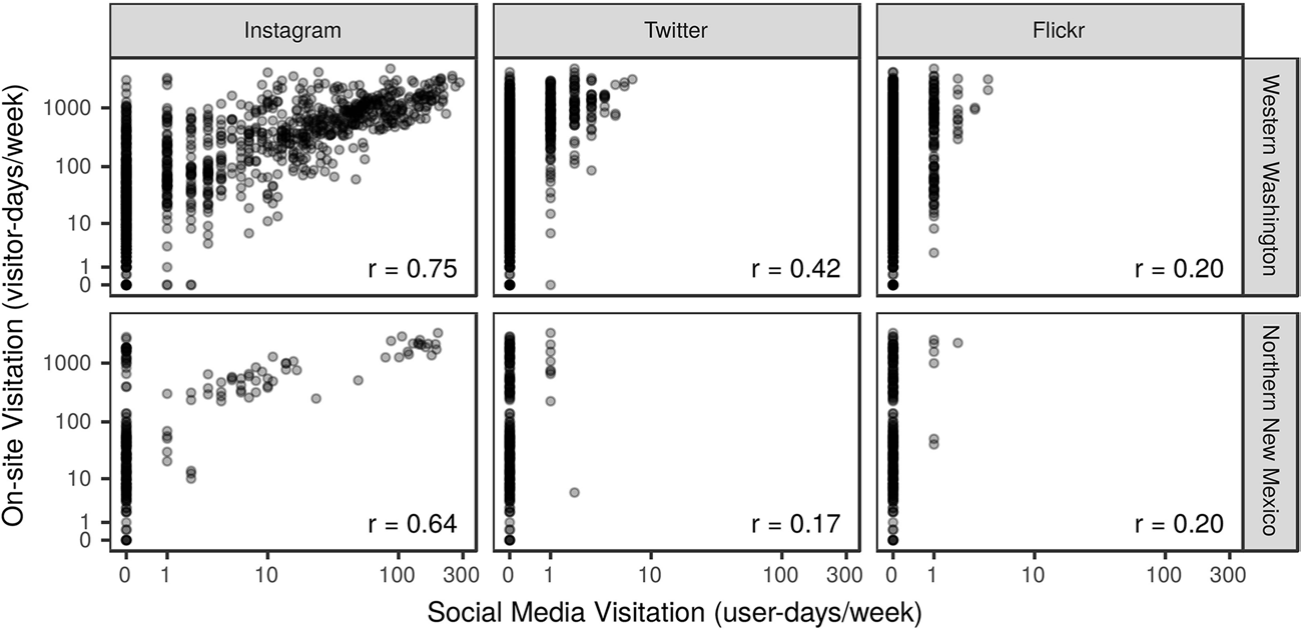
Relationships between observed weekly visitor-days and social media weekly user-days by platform for data collected in WWA in 2016–2018 and in NNM in 2018. Pearson’s correlation (r) between log-transformed on-site counts and log transformed social media user-days is shown in each panel.

We observed relatively strong correlations between weekly visitation and Instagram user-days in both regions. The relationships between observed visitation and both Twitter user-days and Flickr user-days were weaker, but still positive (Fig. 3). All relationships were highly variable, particularly at low levels of visitation when there are few social media posts. Interestingly, we found relatively weak correlations in social media user-days among the social media platforms. The strongest correlation between platforms occurred between Twitter user-days and Instagram user-days (r = 0.52); the relationships between Flickr and Instagram and between Flickr and Twitter were both smaller (r = 0.20 and r = 0.14, respectively).

### Model performance

Using 1,153 site-weeks of WWA data (2016–2018) to model visitation as a function of the calendar, precipitation, and social media variables (Table 2 and Supplementary Table S1), we find that every hypothesized variable is a statistically-significant predictor of on-site visitation (Supplementary Tables S2, S3). The five models give qualitatively similar results regarding the effect of the predictor variables. As expected, weekly visitation is positively related to the number of social media user-days from each platform, holidays, and days of data in each week (our control). On-site visitation is negatively related to weekly precipitation, and shows a strong quadratic relationship to the week of the year (which is correlated with weekly temperature). Model 2 explained approximately 63% of the variability in weekly visitation in WWA (adjusted R^2^ = 0.63, Supplementary Table S2, Supplementary Fig. S2), indicating that it could be improved, potentially by incorporating other predictors or additional on-site counts. Nevertheless, we limited all models to a set of predictors that are readily available for any recreation site nation-wide in the US. The significance of social media predictors from every platform, in combination with the weak correlations among the social media user-days from various platforms, suggests that we gain slightly different information about visitation on public lands from each of the platforms.

**Table 2:** Five approaches (Models 1–5) evaluated as methods for predicting visitation that can be applied depending on whether on-site counts or social media data are available to the researcher or public land manager.

Model	Model details				Scenario	
	Data to build model	Data to test model	Predictors	Effect structure	Access to social media data	Access to on-site counts
1	WWA	2/3 NNM	Calendar + precipitation	Fixed	No	No
2	WWA	2/3 NNM	Calendar + precipitation + social media	Fixed	Yes	No
3	WWA + 1/3 NNM	2/3 NNM	Calendar + precipitation + social media + region	Fixed	Yes	Yes, few sites
4	WWA + 1/3 NNM	2/3 NNM	Calendar + precipitation + region	Fixed and random	No	Yes, many sites
5	WWA + 1/3 NNM	2/3 NNM	Calendar + precipitation + social media + region	Fixed and random	Yes	Yes, many sites

### Predictive power

**Figure 4:**
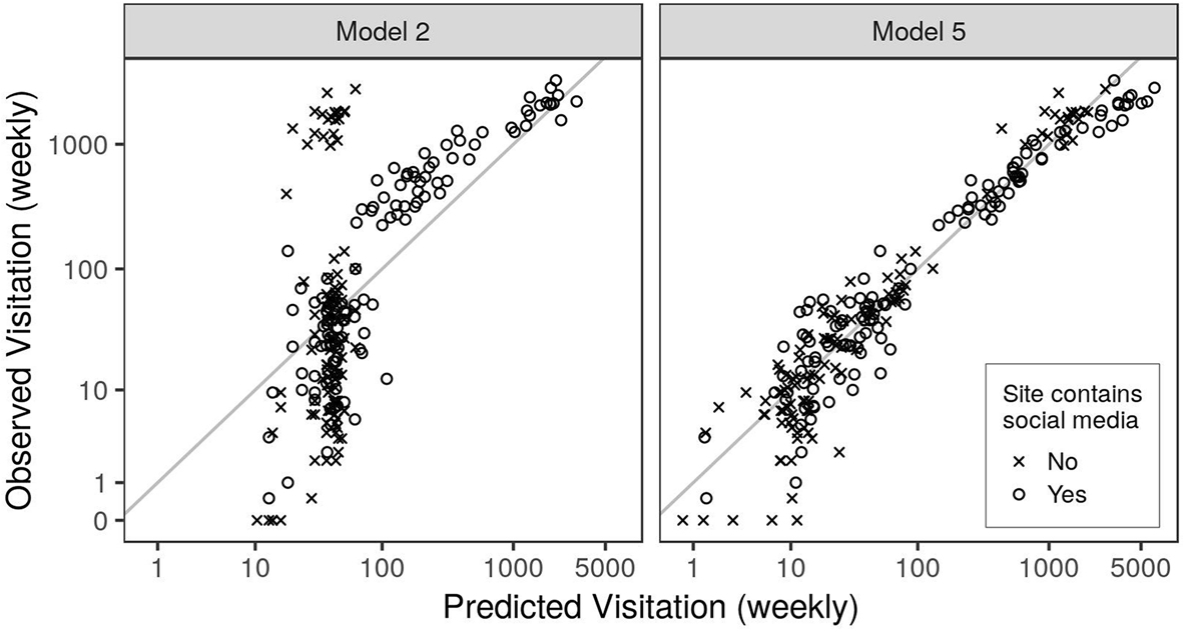
Correlation between observed and predicted visitation at all NNM site-week combinations by Model 2 and Model 5. Predictions by Model 2 are made entirely based on visitation patterns in WWA. Model 5 was fit with an additional random subset of 1/3 of the NNM data and random effects that allow relationships to vary by site. The grey line shows a 1:1 relationship between the variables.

**Figure 5:**
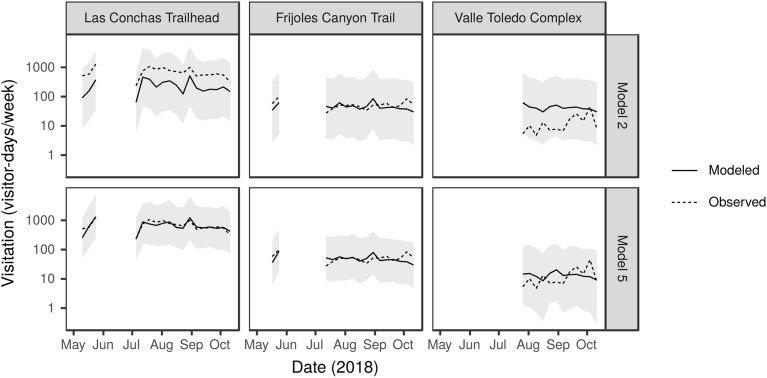
Weekly visitation at three sites in NNM observed via on-site counts and predicted by Model 2 and Model 5. The data are representative of sites with relatively high, medium, and low visitation that contain some social media data. Only weeks with four or more days of on-site counts are shown. Shaded bars show 95% prediction intervals. The 95% prediction intervals for Model 5 include errors from both the fixed and random effects and were calculated using the merTools package in R^50^.

Cross-validation shows that models have differing ability to estimate visitation at sites in NNM that are novel to the model (Tables 2, 3). Model 1, based only on information about how calendar- and weather-related variables explain recreational use in WWA, performs poorly when applied in NNM, as expected, and captures only 6% of the variability in NNM visitation (RMSE = 2.17, r = 0.25, R^2^ = 0.06). Model 2, which adds social media predictors, performs substantially better, capturing 45% of the variability in visitation across all 13 study sites in NNM (RMSE = 1.57, r = 0.67, R^2^ = 0.45). Since Model 2 relies on social media, it performs best at the seven sites that contain some social media and worst at the six study sites where visitors did not share any social media on Flickr, Instagram, or Twitter (Fig. 4). At the seven NNM sites where there are social media data from any time during the study, Model 2 describes 79% of the variability in visitation (RMSE = 0.95, r = 0.89, R^2^ = 0.79). It was most successful at predicting visitation at sites with moderate visitation, and less successful at predicting visitation to sites with especially high or low visitation (Figs. 4, 5).

**Table 3:** Model performance, based on predictions of an out-of-sample subset of 150 site-weeks in NNM across all 13 study sites.

Model	Model performance		
	Mean RMSE	Mean r	R^2^
1	2.17	0.25	0.06
2	1.57	0.67	0.45
3	1.59	0.67	0.45
4	0.64	0.95	0.91
5	0.65	0.95	0.91

Root mean square error (RMSE) and Pearson’s correlations (r) were calculated for each of 500 iterations, then averaged for an overall measure of predictive performance. The coefficient of determination (R^2^) was calculated by squaring the mean correlation, and can be interpreted as the proportion of variance in observed visitation predicted by the model.

Adding 80 site-weeks of observations from NNM into the data used to parameterize the fixed effects model did not result in noticeable improvements in model performance at sites in NNM (Model 3, Tables 2, 3). This model, despite allowing for regional differences in visitation through the incorporation of the “region” factor and being parameterized with data from both study regions, was not able to predict visitation better than a model based solely on data collected in WWA (Model 2). This implies that relationships between precipitation, the calendar, social media posts, and visitation do not differ substantially when the two regions are considered in aggregate.

The further addition of a site-level random effect, on top of 80 site-weeks of NNM observations (Model 4), increased model performance substantially even without variables derived from social media. The predictive power of Model 4 was matched by Model 5, which included social media (Tables 2, 3). Cross-validated predictions by Models 4 and 5 produced a mean correlation of 0.95 between predicted and observed visitation at 150 NNM site-weeks, suggesting that the models account for 91% of the variability in visitation at NNM sites (Table 3, Figs. 4, 5, Supplementary Fig. S2). Model 5 performs equally well when considering only the seven NNM sites that included some social media over the study period (RMSE = 0.60, r = 0.95, R^2^ = 0.91).

## Discussion

This study of recreation in two geographically distinct regions of the United States indicates that social media can predict visitation at recreation sites on public land. We conclude that social media data can be applied with moderate success to estimate visitation at sites that are unmonitored or otherwise lack on-site counts, even in new regions. A basic visitation model that relies solely on generic predictors (e.g., weather, holidays, and seasonality) is only modestly successful due to regional differences in visitor behavior (Model 1). Performance is improved by including relationships between visitation and social media (Flickr, Instagram, and Twitter), even when these relationships are transferred from a different region (WWA, Model 2). These results are consistent with prior research findings that social media counts are correlated with on-site visitor counts from public lands^8,9,24,32^, and extend earlier findings by showing the potential for statistical models to estimate absolute numbers of visitors at unmonitored sites with parameters derived from social media. This is evidence of patterns in how visitors use and share social media. Furthermore, it suggests that measurable variables associated with social media use could support transferable models for accurately estimating visitation to public lands across large geographies.

We expected to detect regional differences in social media use given regional distinctions in climate, land management, population density, mobile phone signal coverage, and the demographics of visitors. Contrary to our expectations, we observed that the rate of posting to social media about recreation visits is similar across sites in NNM and WWA, and both regions displayed positive correlations between each of these social media data sources and observed visitation (Fig. 3). Furthermore, a model parameterized with social media use in WWA (Model 2) explains 45% of the variation in visitation across all 13 sites in NNM and 79% of the variability in visitation at the subset of sites that had social media posts. Re-parameterizing Model 2 with a portion of NNM visitation data does not improve its performance (Model 3) until these data are considered at the site level (Model 5). There is a noisy but consistent relationship between a destination’s popularity with visitors and its popularity on social media, regardless of whether the site is in NNM or WWA. We interpret this as evidence that visitors are equally likely to share their recreation experiences in NNM or WWA, despite the regional differences in the types of recreation opportunities and the people who are visiting.

Although there are consistent relationships between social media use and visitation at a regional scale, we see large site-to-site variability in how visitors use social media within both regions. Beyond simple differences in numbers of posts by site, the proportion of people who post about their visits varies by destination, ranging from 7% of visitors to Kasha–Katuwe Tent Rocks National Monument posting on Instagram to zero recorded social media user-days at several trails in the Valles Caldera National Preserve. As a result, models with random effects that allow sites to have unique relationships between social media, weather, and visitation (Models 4–5) perform substantially better than models that assume social media use is consistently related to visitation for all types of sites (Models 1–3). This is especially true at the most and least visited sites (Figs. 4, 5), where visitors may be sharing social media differently than they do at moderately visited sites, and responding differently to other conditions such as weather or holidays. These results indicate that while data on social media use are helpful for predicting visitation with moderate certainty in an otherwise unknown region (Model 1 vs. 2), their utility for estimating visitation is less clear when local data on the effects of environmental and institutional conditions such as weather and holidays are available to parameterize site-specific models.

A primary goal of this study is to test approaches for estimating visitation over relatively small areas in order to explore the limits of the data and methods. We find that six of the 13 sites in NNM—representing individual trails or groups of trails within a larger park—lack social media during the study period. Model 2, which depends on social media data to estimate visitation at unmonitored sites, consequently performs relatively poorly at these six sites (Fig. 4). Generally, sites that have sparse social media data tend to receive few visitors, but there are exceptions. Alcove House in Bandelier National Monument, for instance, is a very highly visited site that lacks Instagram images in our study because the site does not appear as a prescribed location for Instagram users. Our informal observation is that many visitors instead share untagged photographs of Alcove House or assign their images to other relevant place names such as Bandelier National Monument. Clearly, there are thresholds to where and how social media can be leveraged for visitor estimation. Our research suggests that future studies and visitation models could be improved by accounting for the popularity of sites on social media in the study design^12^. Visitation models that include predictors derived from social media (Models 2, 3, and 5, here) will likely out-perform alternative models for estimating use at popular sites or when longer time series are available. At locations with low or no posting activity, where social media contributes less to visitor estimates, it could be more useful to collect on-site data such as vehicle and pedestrian counts. Further research is necessary to understand what combination of on-site, social media, environmental, and other data is most valuable at different spatial and temporal scales.

These observations suggest that variability in correlations between social media and on-site visitor counts seen here and in previous studies^8,12,20^ is derived from local factors influencing visitors’ day-to-day decisions about whether and how to share a destination on social media. Choices about whether to post to social media are likely influenced by the characteristics of the local site—perhaps its topography, amenities, predominant activity, or unique natural features—and the ways that people relate to these features^33^. The characteristics of the visitors and the relative contributions of the natural versus the social experience in the motivation for the trip may play a role. For example, if a given type of site attracts visitors wishing to “unplug” and have a nature-based outing (a calm forest glade, say), there may be fewer posts per visit than for a site attracting visitors who desire a social experience within a natural setting (a famous scenic overlook, for instance). Another possibility is that the prevailing popularity of certain destinations on social media creates a positive feedback, whereby new visitors feel compelled to share content about their visit in response to the posts of others or the local hashtags that may make it easier or more enticing to post. Variability could also arise from the recent trend towards discouraging visitors from posting geolocated content and attracting attention to less popular or back-country sites that are not equipped to sustain higher use, although this is probably of minor importance, currently.

This is the first study to our knowledge that develops and tests models for estimating absolute numbers of visitors at unmonitored recreation sites or times using multiple social media data sources with differential effects. Building on earlier research exploring relationships of park visitation with numbers of posts to multiple social media platforms^9,12,20,27^, the present study tests whether models with a mixture of predictors to represent varying effects of three online platforms can estimate visitation in novel situations. We find that each social media data source contributes information that explains a statistically significant portion of the variability in visitation and improves the accuracy of the estimate. This is the case not only for Instagram, which captures 3–4% of visitor-days at our research sites in WWA and NNM, but also for Flickr and Twitter, with relatively small amounts of content shared (< 1% of visitor-days). Additionally, while the user-days of content shared on each platform is correlated with actual use at our research sites (Fig. 3), posting frequencies for the three social media platforms are only weakly correlated with each other. Perhaps this is because each platform represents different groups of users who are participating in different recreational activities or differ in their propensity for sharing content about certain types of experiences on public lands. This indicates to us that visitation models could be improved by adding predictors derived from social media platforms beyond these three (historically) popular social media platforms that we chose to test. Social media from platforms that are oriented towards specific types of activities or audiences—such as anglers or bicyclists—who may be under-represented on Flickr, Instagram, and Twitter could be especially valuable^34,35^.

While our understanding of when and how social media are effective for understanding visitor use continues to improve, there are still many unanswered questions and potential issues to consider. The future use of similar approaches will require researchers and practitioners to continually reassess and validate the underlying patterns and assumptions, following best practices for model-building and lessons learned from previous research, such as the failure of Google Flu Trends^36^. Social media users, and those who choose to share geolocated content from parks, are not a representative sample of visitors in most locations^22^. Over time, visitors will likely change how they use social media, including their willingness to geotag and share content, and which online platforms or media are vogue. Furthermore, social media platforms are continually being redesigned in ways that affect the type of content that is available (e.g., images versus text), how it is shared or promoted, and whether researchers and users have access to the information. In 2016, for example, Instagram stopped sharing the geocoordinates that are uploaded by its users^29^. Our study relies entirely on counts of photographs that were assigned by users to prescribed places. These realities must factor into plans for using social media as data now and into the future.

### Practical guidance and implications for managers

The quantity, apparent fine spatial resolution, and “live” nature of social media data make it an enticing information source for recreation practitioners, who often desire knowledge of recreation use and visitor characteristics at fine spatial and temporal resolutions. Traditional approaches rely on visitor intercept surveys and physical technology, such as tube or infrared counters. Based on the results described here and in other research^11,12,17^, we conclude that social media data and related visitation models hold promise for estimating recreation use, especially for large recreation site complexes or collections of recreation resources (e.g., multiple trails within a wilderness area) that are otherwise difficult to monitor. Additionally, social media and other volunteered geographic information provide an opportunity for managers to demonstrate that their recreation monitoring approach is current, practical, and up-to-date with technology.

Previous research has shown that social media data are appropriate for depicting the relative magnitudes of recreation use across a given group of recreation sites. This allows managers to determine when one specific location receives more use than another location^6,8,11^. For estimating the absolute number of visits to a recreation resource, our results suggest that visitation models are currently most effective when some recreation count data are gathered on-site. Our visitation models were able to explain 91% of the variability in actual use at sites in NNM when we included on-site count data, compared with up to 79% when we included only social media, precipitation, and calendar data. For practitioners, this suggests that social media data do not fully substitute for traditional on-site counts. Clearly, social media provide the greatest capacity for estimating recreation use at sites where visitors share at least some content. Social media data may also be most beneficial for filling in spatial and temporal gaps in traditional recreation monitoring programs, to capture unique events or other situations that might cause visitation to deviate from the long-term trend. In addition, these two approaches could be paired to strategically inform one another. For instance, social media data might be used to help stratify individual sites according to relative use (e.g., high, medium, low) for traditional recreation monitoring programs that employ stratified on-site sampling schemes^37,38^.

Considerable technical sophistication is required to gather data from social media platforms and other sources, to aggregate and query data, and to apply statistical models to estimate visitation. This study relies on a mixture of recreation data sources spanning a range of large areas and time periods. Storing, aggregating, and properly querying data required us to develop a data ontology for tracking the location and provenance of various types of visitor counts. Further, multiple types of on-site counts as well as numbers and locations of social media posts were stored with a relational model that could be queried and processed to generate visitation estimates by site and day. Recreation managers likely lack the time, access to computing and storage, and quantitative skills necessary to perform these tasks. This highlights the need for continued partnerships among scientists, practitioners, and data providers to develop tools that practitioners can use to apply new approaches for recreation monitoring programs.

### Next steps for researchers

This study extends our knowledge of how social media can improve estimates of recreation use on public lands. Yet there is a clear need for additional research in other landscapes where visitor demographics and recreation resources differ from those in our two study regions, including urban parks, dispersed-use wilderness, or shoreline areas, and in regions outside of the US. Future research could also apply our current understanding of measuring recreation use with social media data to address questions that currently face recreation managers and policymakers. How, for instance, does recreation use change around sites that are suddenly closed because of management actions or natural disturbance, such as wildfire or flooding? During this study in NNM, multiple sites were closed to the public at various times because of heightened fire risk, active fires, and flooding. It would be interesting to apply the new visitation modeling techniques to investigate if use was redistributed to other substitute sites or whether people ignored certain closures. Alternatively, well-enforced closures might present opportunities to investigate the amount of social media that is falsely or mistakenly posted from locations at times when people are not actually present. Another promising future research topic is the potential feedback loops (positive or negative) between the popularity of social media posts for individual recreation sites and recreation use at those sites, and how managers might anticipate, react to, or mitigate that feedback.

The social media environment is characterized by dynamic popularity of online platforms and evolving data availability and cost. The research community working in this area may benefit from additional research to improve our understanding of how to identify and incorporate new data platforms in existing processes and tools, and address data gaps across time or space caused by changes in data availability. Future research could also consider the possibility that visitors may share the same content on multiple social media platforms. While this study focused on simply counting social media posts to estimate recreation use, other information included in posts clearly offers opportunities for understanding visitation, recreation behavior, and visitor characteristics. The content (words and images) and other metadata associated with social media (such as the user’s profile) may provide additional insights into visitor numbers, experiences, activities, and satisfaction during the recreation visit^39,40^, as well as the characteristics of the visitors themselves, though research is needed in order to understand this potential. Layered on top of these research opportunities is an ongoing need for appropriate protocols to protect individual privacy and maintain ethical standards.

## Methods

### Study area

#### Regions

This study focuses on recreation areas located in the Jemez Mountains in rural Northern New Mexico (NNM) and in the Mt. Baker-Snoqualmie National Forest (MBSNF) in Western Washington (WWA; Fig. 1). In both regions, recreation occurs year-round, however, the geography and ecology differ greatly between WWA and NNM, and the landscape likely shapes differing patterns of visitation. Due to geography and terrain in Washington’s Cascade Mountains, many individual trails and roads are confined to single drainages, resulting in patterns of in-and-out visitation, with visitors returning by the same route they used to enter. Conversely, in NNM, with its high desert plateaus and canyons, trails and roads tend to connect and intertwine, leading to greater dispersed travel across the landscape and between recreation areas. Additionally, there are regional differences between WWA and NNM in terms of the surrounding populations and urban development. While the MBSNF is in close proximity to the large metropolitan population of Seattle, this region of NNM was selected, in part, due to its contrasting rural surroundings. This provides an opportunity to investigate differences in social media use on different types of public lands, and differences in choice of social media platforms between rural and urban populations. These differences between study regions provide opportunities to test how models developed for a longer-term study in the MBSNF^17^ perform with data collected from a new, contrasting location.

#### Public land units

Three federal agencies manage the majority of the public lands in NNM: the Bureau of Land Management (BLM), US Forest Service (USFS), and National Park Service (NPS). Specific management units were selected in partnership with land managers based on their needs and challenges. After these conversations, four units were selected in the NNM area: the Jemez National Recreation Area in the Santa Fe National Forest (USFS), Valles Caldera National Preserve (NPS), Bandelier National Monument (NPS), and Kasha-Katuwe Tent Rocks National Monument (BLM). During the study period, we also continued data collection in WWA in the four ranger districts (Mt. Baker, Darrington, Skykomish, and Snoqualmie Ranger Districts) of our existing study area in the MBSNF^17^ (Fig. 1).

#### Study sites

We collected on-site data at 42 sites that were conceptualized as spatially-distinct recreation destinations in National Forests, National Preserves, or National Monuments in NNM (13 sites) and WWA (29 sites). The sites were selected randomly from a catalog of all recreation destinations in the region, stratified by manager-perceived recreation use level, and independent of the volume of social media posted from the site, as described in Fisher et al.^17^. Six sites in WWA were added to the study in 2017, and seven more were added in 2018 because managers had specific interests. All sites were delineated following methods established by Fisher et al.^17^. We worked with site recreation managers and field staff to spatially define study site boundaries that captured common visitor trip routes. A site—which might include multiple trails, roads, peaks, lakes, cultural sites, or other popular landmarks—represents the area over which a visitor might traverse in under one day. The mapped polygons were digitized in a geographic information system (GIS).

### Study period

Visitation was monitored between August 2016 and November 2018 in WWA and between April and October 2018 at most sites in NNM. Valles Caldera National Preserve was monitored between July and October 2018 in order to meet requirements of a research permit. Researchers visited sites at least once a month during snow-free months to maintain automated counters and collect the on-site counts described below. Three of the study sites in WWA were not sampled in 2018 because of road closures or other logistical challenges in accessing the sites. Six of the NNM sites were temporarily closed from June 1 to July 9, 2018 because of heightened risk of fire. Additionally, Kasha-Katuwe Tent Rocks National Monument was closed due to flooding from August 11–23, 2018. Closures were enforced by gates and physical barricades or by rangers, fire crews, and law enforcement officers stationed at road entrances and patrolling the area. We excluded social media and on-site counts from the analyses during these time periods for these sites.

### On-site estimates

We estimated the number of on-site visitor-days (the number of unique visitors to a site on a given day) following the exact methods described by Fisher et al.^17^, unless otherwise noted. At each site we installed either a passive infrared pedestrian counter (38 sites) or a time-lapse video camera (4 sites). As in Fisher et al.^17^ the counters were installed at the first narrow point in a trail and hidden in trees or rocks to capture normal visitor behavior. A recreation count with a date and time stamp was recorded each time the infrared beam was broken. If more than one person could walk abreast on the trail in front of the sensor, then the recorded counts were calibrated by observing the correct number of passersby for a minimum of 20 min. Cameras were deployed at four sites in the Valles Caldera National Preserve in NNM where trails were deemed too wide to get a reliable count using an infrared counter. They captured images at five- to ten-second intervals during daylight hours. All images were then processed using custom software for detecting motion by objects in the images^17,41^. Visitors in the images flagged by the software were counted manually. Multiple cameras were installed at two of the four study sites which had more than one access point.

Raw counts were converted into estimates of site visitor-days by dividing each calibrated count by two. For the two sites with multiple access points, the counts from the individual cameras were added together after dividing by two. Zero-counts were added to all dates for which no counts were recorded and periods of time during which a site was closed to the public. All data were examined, and faulty or questionable data and site closure dates were removed before analysis. We call these estimates “on-site counts” and believe that this is our most reliable estimate of visitation to each site, while acknowledging that some uncertainty is inherent in these methods^17^.

### Social media estimates

Social media postings shared publicly on Flickr, Instagram, and Twitter were used to predict visitation to the study sites. We calculated the number of user-days (unique social media users who posted each day) per study site for each of the three social media platforms, based on the date and location where the content was created^8^. Data included all publicly available posts that were tagged to a location within the site boundary^8,11,17^. Flickr and Twitter users have the option to share the latitude and longitude where the content was created—using coordinates of the GPS unit in their phone or camera—or to assign a location manually^42^. Flickr images were retrieved from November 16–25, 2018 by querying the Flickr application programming interface (API). Tweets were retrieved in real-time from Twitter’s “statuses/filter” streaming API. We queried both platforms for all geolocated posts that users shared during the study period and were assigned to a location within the study regions (according to Flickr’s “lat” and “lon” objects and Twitter’s “coordinates” object). To geolocate their Instagram images, users only have the option to tag content with a place-name at the time that it is uploaded. To find content on Instagram that was associated with study sites, we manually searched for Instagram places with corresponding names and locations. Some study sites were associated with more than one Instagram place, since sites can contain multiple trails or major landscape recreation destinations, while other study sites were not associated with any Instagram places. Then, from November 14–18, 2018, we recorded the owner and date of every image that was shared on the webpage for each Instagram place during the study period. The spatial distribution of social media posts is shown for both of our study regions in Fig. 1.

### Analytical methods

We created statistical models of weekly visitation using data from the 42 sites in our study regions. The visitation estimates derived from on-site counts were modeled as a function of social media and several additional variables controlling for weather and social factors known to influence visitation (Supplementary Table S1)^43,44^. To control for seasonal differences in visitation, we included a “week of the year” variable, and a dummy variable for whether or not a holiday fell within the modeled week. We also incorporated daily total precipitation from the nearest available NOAA weather station^45^. We considered including daily temperature as a predictor, but found that at a weekly scale it was highly correlated with our calendar variable and so we excluded it. A wide variety of other weather and climate variables such as cloud cover and wind speed have been used by others to explain recreation behavior^46,47^. Because our study focused on the contribution of social media data, model transferability, and potential applications by recreation managers, we constructed the most parsimonious model with only readily-available weather variables that had clear influence on recreation patterns in our study regions. In the interest of model transferability, we chose covariates that should be available for any recreation site in the US.

### Data manipulation

All data were aggregated to a weekly scale, resulting in total visitor-days per week estimated from on-site counts, total weekly user-days of social media per platform, total weekly precipitation, and whether or not a holiday occurred in a given week. Weeks spanned Thursday to Wednesday so that each week contained a single weekend. Due to the administrative closures and occasionally faulty infrared counters or cameras, not all sites had on-site counts for every day of every week. We treated these site-date combinations as missing and excluded any social media or precipitation data from those days. This meant that some estimates of weekly visitation included fewer than seven days of data, so we also included a controlling variable, “days in week”, to describe how many days went into the aggregated estimates. In total, the resulting dataset included 1,153 site-weeks with at least one on-site observation from WWA (2016–2018) and 230 site-weeks from NNM (2018), for a total of 1,383 site-weeks of data.

We applied a natural log transformation to on-site counts, precipitation, and user-days per week from each social media platform in order to better approximate normality. To account for zeros, we added 1 to each variable before log-transforming. In addition, since we expected visitation to be highest in the summer months, we included a quadratic term for week of the year. We also included a factor for region in order to account for any differences in magnitude of visitation between NNM and WWA. We found no indication of multicollinearity in our variables (pairwise correlations all below 0.5 and variance inflation factors all below 2).

### Models

We built five linear models in order to test several competing hypotheses about the importance and relative value of incorporating social media data into visitation models. The models were parameterized using different combinations of our available data (Table 1 and Supplementary Table S1). As such, they represent different scenarios under which researchers and land managers could utilize social media based on data availability. The first two models assume that a land manager has no local visitation data with which to parameterize a model, so they rely on previously collected data from other regions. Model 1 only requires information on precipitation and calendar variables, while Model 2 requires that they also have access to Instagram, Twitter, and Flickr user-day information. Therefore, these models quantify how much of the variability in visitation can be explained by calendar and precipitation variables alone (Model 1), and with the addition of social media user-day variables (Model 2). Model 3 assumes that land managers have access to some on-site counts from their region, in addition to calendar, precipitation, and social media data. This model was parameterized using 80 site-weeks of data from NNM (approximately 1/3 of the NNM data) in addition to all the WWA data, in order to allow for regional differences in the relationship between social media use and visitation rate. These 80 site-weeks were a random subset of the 230 total site-weeks of data from NNM. We reserved the remaining 150 site-weeks as “out-of-sample” data for model testing (below). Models 1–3 are linear fixed effect models,

1 $$\begin{array}{*{20}c} {ln\left( {Y_{it} } \right) = \alpha + {\bf x}_{it}^{T} \varvec {\beta} + \varepsilon_{it} } \\ {\varepsilon_{it} \sim Normal\left( {0,\sigma^{2} } \right),} \\ \end{array}$$

where $Y_{it}$ is the number of visitors observed at site $i$ during week $t$, ${\bf x}_{it}^{T} = \left( {x_{it1,} x_{it2} , \ldots ,x_{itp} } \right)$ are the values of variable $p$ at site $i$ during week $t$, and $\varvec {\beta}^{T} = \left( {\beta_{0,} \beta_{1} , \ldots ,\beta_{p} } \right)$ is a vector of coefficients. Models were fit with R using the lm function^48^. Models 4 and 5 are linear mixed models which incorporate all of the fixed effects listed above as well as a random site-level effect (Table 1 and Supplementary Table S1). These models explicitly incorporate terms to account for differences among recreation sites, and thus require that managers have access to on-site data from the majority of sites they wish to study, in addition to precipitation and calendar data. Model 5 also requires social media data, while Model 4 does not. We parameterized these models with a random subset of the NNM data (80 of 230 weeks, as in Model 3) in a linear mixed model,

2 $$ \begin{array}{*{20}c} {ln\left( {Y_{it} } \right) = \alpha_{i} + {\bf x}_{it}^{T} \varvec {\beta} + \varepsilon_{it} } \\ {\alpha_{i} \sim Normal\left( {\mu_{\alpha } ,\sigma_{\alpha }^{2} } \right)} \\ {\varepsilon_{it} \sim Normal\left( {0,\sigma^{2} } \right),} \\ \end{array} $$

where $Y_{it}$, ${\bf x}_{it}^{T}$, and $\varvec {\beta}^{T}$ are as defined above, and $\alpha_{i}$ is a random site-level effect at site $i$. Mixed models were fit using the lme4 package in R^49^.

### Model testing

We used out-of-sample predictive power as our measure of model performance, since we are primarily interested in addressing questions about the generalizability of visitation models. The out-of-sample dataset was always a random 150 site-weeks of NNM data which were not used to parameterize the models (approximately 2/3 of the NNM data). For each of the five model specifications (Table 1), we randomly generated an out-of-sample dataset, fit the model, predicted visitation for the out-of-sample week/site combinations, calculated the root mean square error (RMSE) of the predictions vs. observed values, and calculated the Pearson’s correlation (r) between the predicted and observed values. This process was repeated 500 times for each model. From the 500 iterations, we calculated the mean RMSE and mean correlation for each model, then squared the resulting mean correlation to find the coefficient of determination (R^2^).

## Supplementary information

Supplementary Information.

## Data Availability

The datasets analyzed here are available from the corresponding author by request.
