# Supplementary material for: Next-generation visitation models using social media to estimate recreation on public lands
Source: Sci Rep. 2020 Sep 22;10:15419. doi: 10.1038/s41598-020-70829-x (PMC7508982; doi:10.1038/s41598-020-70829-x)
Supplement: Supplementary file 1 — Supplementary Information. [file 41598_2020_70829_MOESM1_ESM.pdf]

Supplementary Information

Next-generation Visitation Models using Social Media to Estimate Recreation on Public Lands

Spencer A. Wood[1,2], Samantha G. Winder[2], Emilia H. Lia[2], Eric M. White[3], Christian S.L. Crowley[4], Adam A. Milnor[5]

[1] eScience Institute, University of Washington, Seattle, WA US
[2] EarthLab, University of Washington, Seattle, WA US
[3] Pacific Northwest Research Station, US Forest Service, Olympia, WA US
[4] Office of Policy Analysis, US Department of the Interior, Washington, DC US
[5] Rivers, Trails, and Conservation Assistance Program, National Park Service, Tucson, AZ US

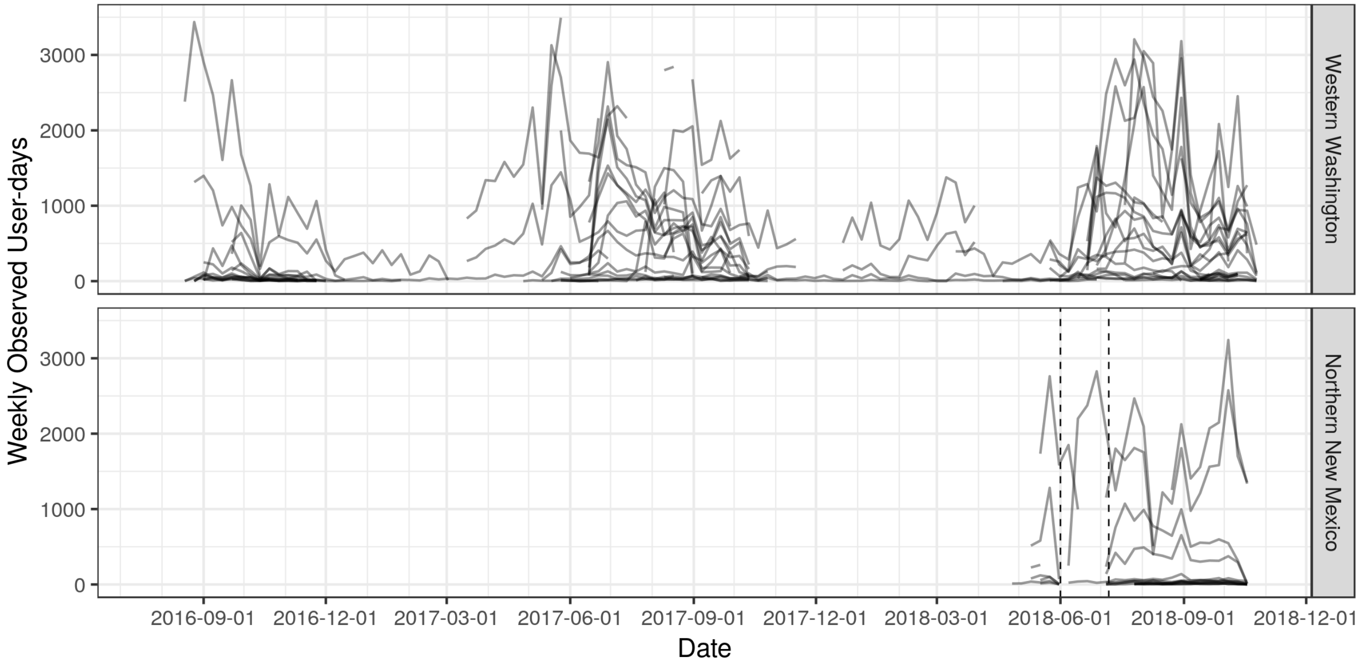

Supplementary Figure S1. Weekly visitation by site. Dashed lines indicate the period of a fire-related closure in Northern New Mexico. Other gaps in the lines represent periods when sites were closed to the public or on-site counts were not collected.

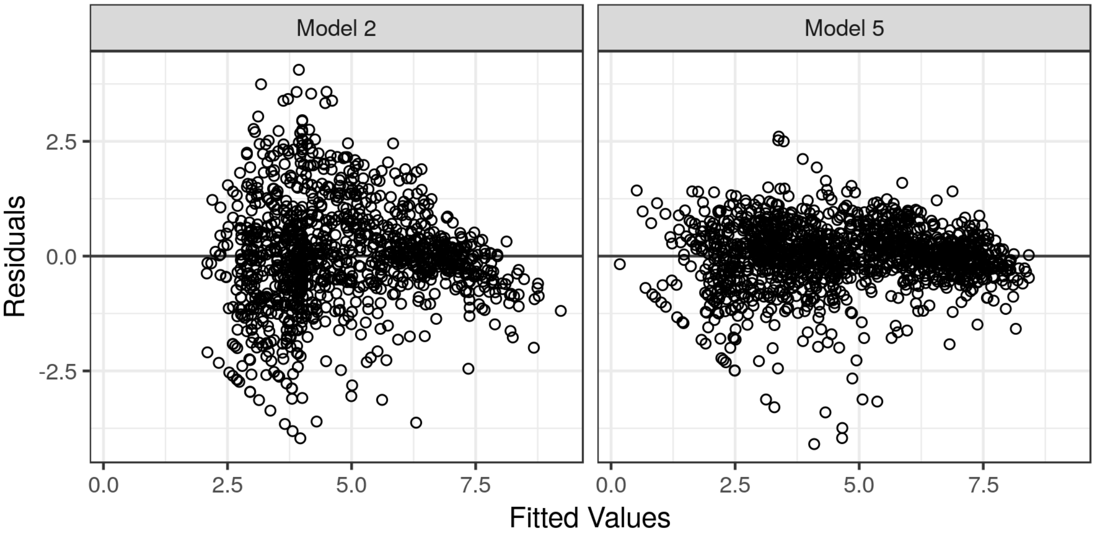

Supplementary Figure S2. Pearson's residuals (y axis) plotted against the fitted values (x axis) for Models 2 and 5.

Supplementary Table S1. Variables used to parameterize Models 1-5 of weekly visitation in Western Washington (WWA) and Northern New Mexico (NNM).

Variable	Description	Units	Median	Minimum	Maximum	Transformation
**Calendar**						
week of year	ordered week		34	1	52	scaled 0-1, then squared, with linear and quadratic terms included in model
holiday	holiday in week	binary	0	0	1	
**Weather**						
precipitation	total precipitation	inches	0.13	0	4.45	natural log
**Social Media**						
Instagram		user-days	1	0	283	natural log
Twitter		user-days	0	0	7	natural log
Flickr		user-days	0	0	4	natural log
**Region**						
region	WWA or NNM	categorical				
**Days in Week**						
days in week	control	days	7	1	7	

Supplementary Table S2. Coefficient estimates for Model 2, a linear fixed effects model describing the relationship between weekly on-site counts (natural log transformed) and calendar, weather, and social media posts at 29 sites in Western Washington between 2016 and 2018. Adjusted R^2 = 0.63.

Variable	Coefficient	Standard Error	t-value	P value
intercept	1.223	0.282	4.342	<.0001
days in week	0.207	0.030	6.963	<.0001
week of year	4.946	0.795	6.218	<.0001
$(\text{week of year})^2$	-4.591	0.738	-6.221	<.0001
holiday	0.227	0.103	2.212	0.027
ln(precipitation)	-0.559	0.010	-5.609	<.0001
ln(Flickr user-days)	0.499	0.144	3.457	<.001
ln(Twitter user-days)	0.320	0.104	3.075	0.002
ln(Instagram user-days)	0.714	0.023	31.165	<.0001

Supplementary Table S3. Coefficient estimates for Model 5, a linear mixed effects model describing the relationship between weekly on-site counts (natural log transformed) and calendar, weather, and social media posts at 29 sites in Western Washington (WWA) between 2016 and 2018 and 13 sites in Northern New Mexico (NNM) in 2018. This model includes a site-level random effect (variance = 1.861, SD = 1.364) and uses all of the data collected in NNM. P-values are not calculated for this model because they are not well defined for mixed-effects models[49].

Variable	Coefficient	Standard Error	t-value
intercept	0.590	0.419	1.407
days in week	0.345	0.016	21.078
week of year	4.997	0.536	9.319
$(\text{week of year})^2$	-4.907	0.482	-10.189
holiday	0.381	0.059	6.472
ln(precipitation)	-0.643	0.059	-10.826
ln(Flickr User-days)	0.219	0.089	2.470
ln(Twitter user-days)	-0.027	0.068	-0.392
ln(Instagram user-days)	0.291	0.028	10.310
project (WWA)	0.829	0.460	1.800
